# Genome degradation promotes *Salmonella* pathoadaptation by remodeling fimbriae-mediated proinflammatory response

**DOI:** 10.1093/nsr/nwad228

**Published:** 2023-09-02

**Authors:** Xiao Zhou, Xiamei Kang, Jiaqi Chen, Yan Song, Chenghao Jia, Lin Teng, Yanting Tang, Zhijie Jiang, Xianqi Peng, Xiaoxi Tao, Yiwei Xu, Linlin Huang, Xuebin Xu, Yaohui Xu, Tengfei Zhang, Shenye Yu, Jiansen Gong, Shaohui Wang, Yuqing Liu, Guoqiang Zhu, Corinna Kehrenberg, François-Xavier Weill, Paul Barrow, Yan Li, Guoping Zhao, Min Yue

**Affiliations:** Institute of Preventive Veterinary Sciences and Department of Veterinary Medicine, Zhejiang University College of Animal Sciences, Hangzhou 310058, China; Ningbo Academy of Agricultural Sciences, Ningbo 315040, China; Institute of Preventive Veterinary Sciences and Department of Veterinary Medicine, Zhejiang University College of Animal Sciences, Hangzhou 310058, China; Institute of Preventive Veterinary Sciences and Department of Veterinary Medicine, Zhejiang University College of Animal Sciences, Hangzhou 310058, China; Institute of Preventive Veterinary Sciences and Department of Veterinary Medicine, Zhejiang University College of Animal Sciences, Hangzhou 310058, China; Institute of Preventive Veterinary Sciences and Department of Veterinary Medicine, Zhejiang University College of Animal Sciences, Hangzhou 310058, China; Hainan Institute of Zhejiang University, Sanya 572025, China; Institute of Preventive Veterinary Sciences and Department of Veterinary Medicine, Zhejiang University College of Animal Sciences, Hangzhou 310058, China; Institute of Preventive Veterinary Sciences and Department of Veterinary Medicine, Zhejiang University College of Animal Sciences, Hangzhou 310058, China; Institute of Preventive Veterinary Sciences and Department of Veterinary Medicine, Zhejiang University College of Animal Sciences, Hangzhou 310058, China; Institute of Preventive Veterinary Sciences and Department of Veterinary Medicine, Zhejiang University College of Animal Sciences, Hangzhou 310058, China; Institute of Preventive Veterinary Sciences and Department of Veterinary Medicine, Zhejiang University College of Animal Sciences, Hangzhou 310058, China; Institute of Preventive Veterinary Sciences and Department of Veterinary Medicine, Zhejiang University College of Animal Sciences, Hangzhou 310058, China; Institute of Preventive Veterinary Sciences and Department of Veterinary Medicine, Zhejiang University College of Animal Sciences, Hangzhou 310058, China; Department of Microbiology Laboratory, Shanghai Municipal Center for Disease Control and Prevention, Shanghai 200336, China; College of Veterinary Medicine, Henan University of Animal Husbandry and Economy, Zhengzhou 450053, China; Key Laboratory of Prevention and Control Agents for Animal Bacteriosis, Institute of Animal Husbandry and Veterinary, Hubei Academy of Agricultural Sciences, Wuhan 430064, China; Division of Bacterial Diseases, State Key Laboratory of Veterinary Biotechnology, Harbin Veterinary Research Institute, Chinese Academy of Agricultural Sciences, Harbin 150069, China; Poultry Institute, Chinese Academy of Agricultural Sciences, Yangzhou 225125, China; Department of Animal Public Health, Shanghai Veterinary Research Institute, Chinese Academy of Agricultural Sciences, Shanghai 200241, China; Shandong Key Laboratory of Animal Disease Control and Breeding, Institute of Animal Science and Veterinary Medicine, Shandong Academy of Agricultural Sciences, Jinan 250100, China; College of Veterinary Medicine, Yangzhou University, Yangzhou 225009, China; Institute for Veterinary Food Science, Faculty of Veterinary Medicine, Justus-Liebig University Giessen, Giessen 35392, Germany; Institut Pasteur, Université Paris Cité, Unité des bactéries pathogènes entériques, Paris 75724, France; School of Veterinary Medicine, University of Surrey, Guildford GU2 7AL, UK; Institute of Preventive Veterinary Sciences and Department of Veterinary Medicine, Zhejiang University College of Animal Sciences, Hangzhou 310058, China; Hainan Institute of Zhejiang University, Sanya 572025, China; School of Life Science, Hangzhou Institute for Advanced Study, University of Chinese Academy of Sciences, Hangzhou 310024, China; CAS Key Laboratory of Synthetic Biology, Institute of Plant Physiology and Ecology, Shanghai Institutes for Biological Sciences, Chinese Academy of Sciences, Shanghai 200031, China; Department of Microbiology and Microbial Engineering, School of Life Sciences, Fudan University, Shanghai 200433, China; Institute of Preventive Veterinary Sciences and Department of Veterinary Medicine, Zhejiang University College of Animal Sciences, Hangzhou 310058, China; Hainan Institute of Zhejiang University, Sanya 572025, China; State Key Laboratory for Diagnosis and Treatment of Infectious Diseases, National Clinical Research Center for Infectious Diseases, National Medical Center for Infectious Diseases, The First Affiliated Hospital, College of Medicine, Zhejiang University, Hangzhou 310003, China; Zhejiang Provincial Key Laboratory of Preventive Veterinary Medicine, Hangzhou 310058, China

**Keywords:** *Salmonella*, host adaptation, pathogenic evolution, virulence evolution, vertical transmission, fimbrial adhesin

## Abstract

Understanding changes in pathogen behavior (e.g. increased virulence, a shift in transmission channel) is critical for the public health management of emerging infectious diseases. Genome degradation via gene depletion or inactivation is recognized as a pathoadaptive feature of the pathogen evolving with the host. However, little is known about the exact role of genome degradation in affecting pathogenic behavior, and the underlying molecular detail has yet to be examined. Using large-scale global avian-restricted *Salmonella* genomes spanning more than a century, we projected the genetic diversity of *Salmonella* Pullorum (bvSP) by showing increasingly antimicrobial-resistant ST92 prevalent in Chinese flocks. The phylogenomic analysis identified three lineages in bvSP, with an enhancement of virulence in the two recently emerged lineages (L2/L3), as evidenced in chicken and embryo infection assays. Notably, the ancestor L1 lineage resembles the *Salmonella* serovars with higher metabolic flexibilities and more robust environmental tolerance, indicating stepwise evolutionary trajectories towards avian-restricted lineages. Pan-genome analysis pinpointed fimbrial degradation from a virulent lineage. The later engineered *fim*-deletion mutant, and all other five fimbrial systems, revealed behavior switching that restricted horizontal fecal–oral transmission but boosted virulence in chicks. By depleting fimbrial appendages, bvSP established persistent replication with less proinflammation in chick macrophages and adopted vertical transovarial transmission, accompanied by ever-increasing intensification in the poultry industry. Together, we uncovered a previously unseen paradigm for remodeling bacterial surface appendages that supplements virulence-enhanced evolution with increased vertical transmission.

SIGNIFICANCE STATEMENT Elucidating molecular detail associated with the emergence of bacterial virulence is critical to developing tools to predict disease outbreaks and establish effective intervention strategies. A tractable model system could allow us to dissect the ecological, genetic and evolutionary drivers that foster the selection of virulence traits and transmission dynamics posed by modern anthropogenic activities. Here, we used *Salmonella* Pullorum, causing fatal sepsis in young chickens, as a model system to investigate the genetic and ecological forces leading to an emerging pathogen. Our work offers a mechanistic insight into virulence-enhancing evolution by reducing environmental versatility and, more generally, demonstrates how essential fimbrial appendages change pathogenic behavior. We underlined a novel evolutionary enhancement of virulence and transmission in a host-restricted pathogen during agricultural intensification.

## INTRODUCTION

Over the past few decades, we have witnessed an accelerating prevalence, magnitude and intensification of emerging and re-emerging infectious diseases. These are mainly driven by ecological, climatic and anthropogenic parameters across the world [1–5]. The mechanisms by which gene point mutations or gene modifications [6,7] and gene acquisition via horizontal gene transfer [8,9] occur in the development of pathogen evolution have been well documented. Obligately host-restricted pathogens frequently exhibit extensive genome degradation via gene depletion and inactivation as the pseudogene [10,11]. Investigation of the molecular mechanisms and underlying drivers involved in host adaptation is vital for appreciating pathoadaptive evolution and the emergence of infectious disease, and will provide the knowledge needed for designing targeted interventions and disease eradication [12,13].

The virulence of facultative pathogens with broad host niches is believed to be associated with host generalism and genetic plasticity via multi-step horizontal gene transfer events [12,14], while for the obligate pathogen, host-niche specialism and genetic homogeneity are two hallmarks of pathoadaptation or mutualism. Previous investigations suggested that gene depletion and accumulation of pseudogenes play a vital role in *Salmonella* host adaptation [11,15], and a significant number of pseudogenes are enriched in functional categories, i.e. membrane/surface structure and central/intermediary metabolism [6,11,16]. There is an amounting body of evidence for the association between a given pathogen's central/intermediary metabolism and its virulence [17–20]. However, the mechanistic insights between membrane/surface structure depletion of a pathogen and host-adaptive bonding are relatively rarely investigated.

On the surface of the bacterium *Salmonella*, a variety of virulence factors participate as colonization factors, including a range of fimbrial appendages. Importantly, many bacterial appendages, including lipopolysaccharide (LPS), lipoteichoic acid, flagella and fimbriae, could serve as pathogen-associated molecular patterns (PAMPs), triggering the host immune response [21]. Indeed, the immune response hijacked by *Salmonella* effectors has been well documented [22], and a loss of surface appendages, i.e. flagella and fimbriae, seems to be a key feature for *Salmonella* host-restricted evolution, in particular for avian-specific *Salmonella* [23,24]. It remains unknown if the modification of the surface appendages could play a role in bacterial pathogenic behavior. *Salmonella enterica* serovar Gallinarum, an avian-restricted serovar, includes two common variants or biovars, Pullorum (causing Pullorum disease) and Gallinarum (causing fowl typhoid), resulting in devastating damage and economic loss in developing countries, including China [25–29], where increasing antimicrobial resistance (AMR) in these strains has become a significant concern [30–34]. Notably, *Salmonella enterica* serovar Gallinarum biovar Pullorum (bvSP) has a strong preference for affecting newly hatched chicks; in contrast, biovar Gallinarum (bvSG) mainly causes lethal diseases in adult birds [35]. Additionally, bvSP is very poor at colonization and survival in the gastrointestinal tract, and it can further develop transovarial transmission during industrialized breeding, where the underlying evolutionary driver remains obscure. Here, we used the avian-restricted pathogen bvSP as a model to address the questions, and proposed an evolution pathway via functional loss, including a loss-of-genes important for survival outside the host and within the host niche, resulting in enhanced virulence and an optimized choice for vertical transmission efficacy.

## RESULTS AND DISCUSSION

### bvSP ST92 is the geo-temporal dominant in China

**Figure 1.**
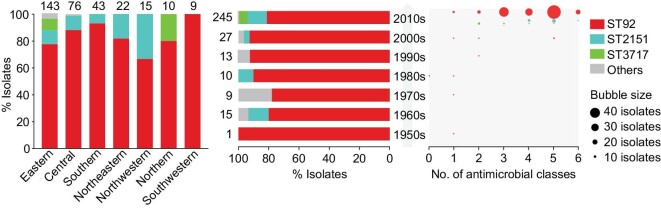
Genetic diversity of *Salmonella* Pullorum by geography, time and multidrug resistance (MDR) rate. Left panel shows the proportion of sequence types of isolates collected in the corresponding regions of China, with the total number of isolates shown above each bar. Two strains of unknown geographic origin are not shown. The middle panel indicates the sequence type distribution of all the isolates collected per decade. The bubble chart shows the distribution of MDR isolates in our laboratory (*n* = 221) per decade. The bubble size indicates the number of MDR isolates. The bubble color indicates sequence type (ST).

To investigate the predominant bvSP in China, a total of 320 isolates, including 221 from our laboratory, were collected between 1954 and 2020 during passive surveillance in local veterinary clinics. In this study we have sequenced 321 new genomics, including 299 bvSP. bvSP has been widespread throughout China, especially in recent years, with the eastern region being the most affected, followed by the central and southern regions. Notably, we found that sequence type (ST) 92 was the dominant type across time (*n* = 265), while ST3717 was newly recovered from northern and eastern China in the past two decades. All isolates were examined in our laboratory for antimicrobial susceptibility against 12 antimicrobial agents of 8 classes, manifesting high-level resistance to 7 antimicrobial agents, particularly to quinolones (215/221). The majority of isolates that were isolated within the past two decades (184/221) exhibited multi-drug resistance (MDR) to up to six antimicrobials (Fig. 1, Supplementary Fig. 1a, b and Supplementary Table 1).

### A recent expansion of bvSP lineages with strong AMR potential

**Figure 2.**
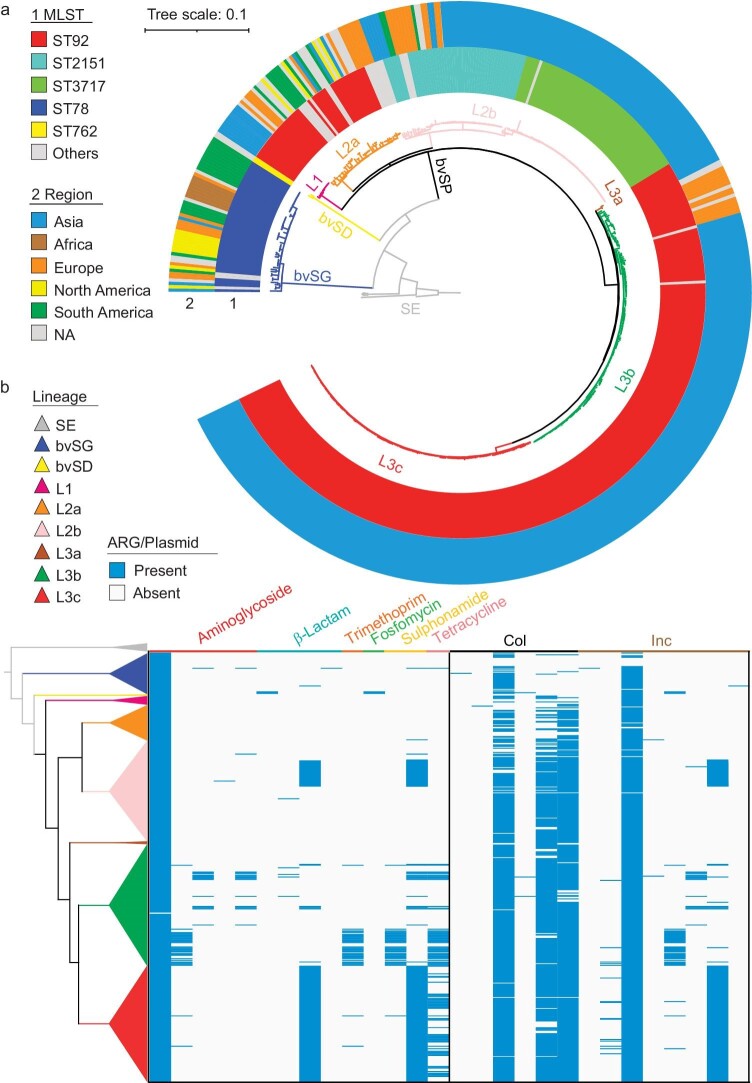
Population genomics of global *Salmonella* Pullorum isolates. The maximum-likelihood phylogenetic tree based on core genome single-nucleotide polymorphisms (SNPs) and bvSP was defined as three lineages. Colored branches show type: *Salmonella* Enteritidis (SE, gray); bvSG (blue); bvSD (yellow); bvSP (black); L1 (rose red); L2a (orange); L2b (pink); L3a (brown); L3b (green); L3c (red). (a) Multilocus sequence types (MLSTs, circle 1) and geographical origin of isolates (continent, circle 2). Minor sequence types are gathered as others. (b) Antimicrobial-resistant gene (ARG) and plasmid patterns. For ease of analysis, plasmids are divided into two groups according to the naming and type. Col refers to plasmids that contain genes coding for bacteriocins, proteins that can kill other bacteria. Inc refers to plasmids classified by the incompatibility (Inc) typing method. Plasmids incompatible with one another are assigned to the same incompatibility or Inc group, while those that can exist together generally belong to different incompatibility groups.

To understand the population structure of bvSP, 458 global bvSP genomes, including 76 non-Chinese strains, were included. Additionally, genetically related bacteria, i.e. *Salmonella* Enteritidis (*n* = 5), bvSG (*n* = 49) and *Salmonella* Gallinarum biovar Duisburg (bvSD, *n* = 2), were also incorporated in the analysis. We obtained the complete genome of bvSP R51 by third-generation sequencing and used it as a reference for projecting a maximum likelihood phylogenetic tree (Fig. 2a and Supplementary Table 2). The phylogenetic relationship between bvSP, bvSG and *Salmonella* Enteritidis is consistent with the previous study. Three lineages of bvSP, along with their respective sublineages, were defined. Based on the phylogeny and metadata, i.e. time, origin, ST, antimicrobial resistance genes (ARGs) and plasmids, lineage 1 (L1) was considered the ancestral bvSP, while lineage 2 (L2) and lineage 3 (L3) were suggested as two branches that evolved in parallel. Most isolates (L1, L2b and L3) originated from China, where L3 was most prevalent (Supplementary Fig. 2), and non-Chinese isolates were mainly grouped in L2a. As in China, the worldwide predominant type was ST92, highly corresponding to L1 and L3 and most of L2a. Sequence types ST2151 and ST3717 were all clustered in L2b. ST3717 was recently detected in China. bvSG, mainly ST78, was observed in Europe, Africa and America. Notably, recent bvSP strains carried more ARGs and plasmids than older strains. The newly evolved L2b and L3c acquired *bla*_TEM-1B_ and *sul2* genes and an IncX1 plasmid (90.97%), as confirmed by the conjugation assay (Fig. 2b). Interestingly, there were no MDR isolates in L1, and MDR isolates pile up only in L2 and L3 (*P* < 0.0001; unpaired *t*-test) (Supplementary Fig. 1c, d). In addition, temporal AMR trends were analyzed (1920–2020), showing a significant correlation between AMR and time among bvSP lineages (*P* < 0.0001) (Supplementary Fig. 1e). Indeed, the recently evolved populations, L3c and L2b, have the most MDR isolates (Supplementary Fig. 1f–h and Supplementary Table 2).

### Enhanced virulence evolution among bvSP lineages

**Figure 3.**
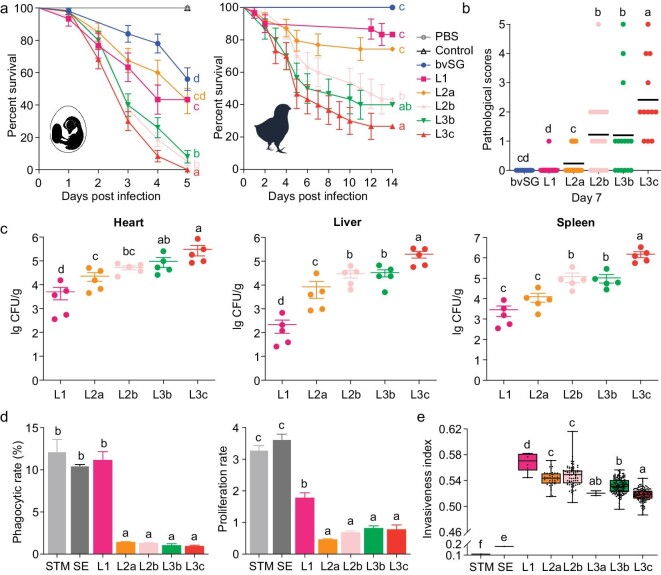
Stepwise virulence enhancing evolution in *Salmonella* Pullorum. (a) The survival curves of chicken embryos (*n* = 30 to 80) and chicks (*n* = 30 to 39) infected with bvSP isolates from each lineage (three to six isolates per lineage in the chicken embryo model, with the same lineage grouping together; one isolate per lineage in the chick model). Uninoculated embryos and inoculation with Phosphate Buffer Saline (PBS) were considered controls. (b) Clinical symptoms of each chick were observed and documented at 7 dpi. (c) The infected chick's heart, liver and spleen samples were harvested and tested for bacterial loads at 7 dpi (*n* = 5). (d) HD11 phagocytosis and intracellular proliferation (12 h) assay for isolates from individual lineages. One isolate per lineage is tested. *Salmonella* Typhimurium (STM) strain SL1344, and *Salmonella* Enteritidis (SE) strain P125109 are used as controls. (e) Lineage distribution of invasiveness index for global bvSP genomes (*n* = 388). STM strain SL1344 and SE strain P125109 are used as controls. Letters a, b, c, d, e and f indicate statistically significant differences of *P* < 0.05 between lineages. Bars with no common letters are significantly different (*P* < 0.05).

To investigate dynamic virulence features among bvSP lineages, isolates selected from each sublineage were examined *in vivo* (with chicken embryos, three to eight isolates per sublineage or with a chick model, one isolate per sublineage) and *in vitro* (HD11 cells, one isolate per sublineage). L1 showed the lowest virulence (56.67% of chicken embryos, 16.67% of chick), while L3c (100% of chicken embryos, 73.33% of chick) and L2b (95% of chicken embryos, 56.67% of chick), the recently evolved populations, had the highest death rate (chicken embryos model: *P* < 0.001, chick model: *P* < 0.01; Log-rank Mantel-Cox test), clinical symptom score (*P* < 0.0001; unpaired *t*-test) and bacterial load in tissues (*P* < 0.0001; ordinary one-way ANOVA). Considering the sampling dates, we detected a virulence-promoting pathway in bvSP. When studying L2, we found the recently evolved L2b was more virulent than L2a (chicken embryo death rate: *P* < 0.0001, chick death rate: *P* = 0.0179, clinical symptom score: *P* = 0.0003, bacterial load in tissues: *P* = 0.0638 for heart, *P* = 0.0409 for liver, *P* = 0.0082 for spleen). And the same phenomenon was observed in L3, namely that L3c was more virulent than L3b (chicken embryo death rate: *P* = 0.0149, chick death rate: *P* = 0.3533, clinical symptom score: *P* = 0.0413, bacterial load in tissues: *P* = 0.0901 for heart, *P* = 0.0136 for liver, *P* = 0.0005 for spleen) (Fig. 3a–c and Supplementary Table 3). Interestingly, L1 behaved similarly to broader host-promiscuous serovars, i.e. *Salmonella* Typhimurium and Enteritidis (*P* = 0.9370 and 0.9876, respectively; ordinary one-way ANOVA), while a significantly higher level of anti-phagocytic capacities against chicken macrophages (*P* < 0.0001) and a relatively low level of intracellular replication potential (*P* < 0.01) were observed for recently evolved lineages in bacteria–macrophage interactions, which suggested a stepwise host adaptation (Fig. 3d). Additionally, the *in silico* analysis with the invasiveness index further supported increased virulence during bvSP evolution in young chicks but not older chickens (Fig. 3e and Supplementary Table 4).

### Loss of environmental flexibility may select for host-restricted adaptation

**Figure 4.**
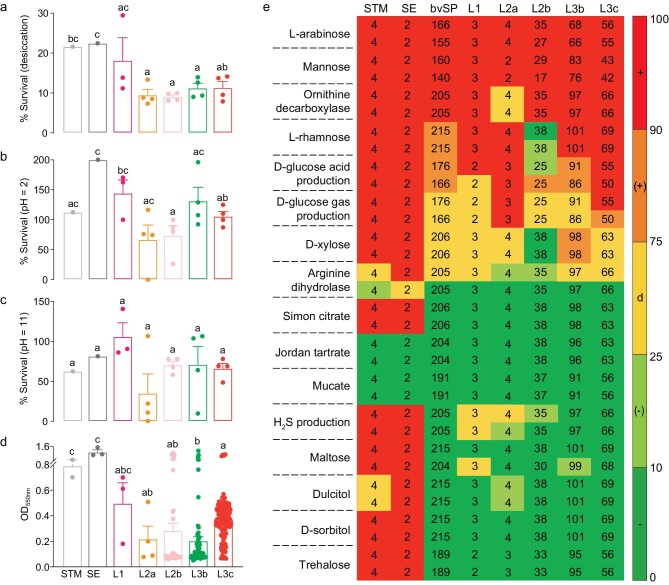
The potential for environmental capabilities among different lineages. (a–c) The survival rate of isolates from different lineages under desiccation (a), acid (pH = 2) (b) and alkali (pH = 11) (c) stress conditions (*n* = 3 or 4). (d) Biofilms of 228 bvSP isolates from different lineages are tested by statical growth in Trypticase Soy Broth (TSB) at 28°C for 48 h. (e) Biochemical phenotypes of different lineages were evaluated towards 16 biochemical tests (list on the left) under aerobic (the first row corresponding to each compound) and anaerobic (the second row corresponding to each compound) conditions. The number in each cell represents the number of isolates tested. The color of individual cells varies with the percentage of compound utilization. Red, +, 90%–100% positive; orange, (+), 76%–89% positive; yellow, d, 26%–75% positive; light green, (-), 11%–25% positive; green, -, 0%–10% positive. STM and SE isolates are used as control strains (*n* = 1 to 4). Letters a, b and c indicate statistically significant differences of *P* < 0.05 between lineages. Bars with no common letters are significantly different (*P* < 0.05).

Next, we examined bvSP survival under environmental stress conditions (*n* = 3 or 4), i.e. desiccation, acid and alkali, and biofilm-forming capabilities (*n* = 228) to investigate the adaptability of bvSP lineages under an outside-host environment. We found that the survival and biofilm formation of recently evolved lineages were weaker than those of ancestral L1 (respectively compared L1 with L2a, L2b, L3b and L3c: desiccation: *P* = 0.1458, 0.1162, 0.2233 and 0.2385, acid: *P* = 0.0734, 0.0464, 0.7097 and 0.1235, alkali: *P* = 0.0795, 0.0732, 0.3052 and 0.0603, biofilm: *P* = 0.1840, 0.3600, 0.0717 and 0.3120; unpaired *t*-test) (Fig. 4a–d and Supplementary Tables 5, 6), and L1 isolates showed similar results to the environment-prone *Salmonella* Typhimurium and Enteritidis (respectively compared L1 with *Salmonella* Typhimurium and Enteritidis: desiccation: *P* = 0.7921 and 0.7433, acid: *P* = 0.5493 and 0.3374, alkali: *P* = 0.3408 and 0.5580, biofilm: *P* = 0.1992 and 0.2022) [36], suggesting that the ability to survive in the environment was reduced during bvSP host adaptation.

To evaluate the full spectrum of biochemical activity of bvSP, we performed 16 biochemical tests under aerobic conditions. The biochemical pattern of L1 was markedly different from that of L2 and L3. Critical tests to distinguish L1 from L2 and L3 were citrate, tartrate and mucate utilization, and H_2_S production, with positive results for L1 and negative results for L2 and L3 (Fig. 4e and Supplementary Table 7). We compared the overall ability of individual lineages to utilize these compounds. The utilization rate of L1 was higher than that of L2 and L3 and closer to that of environmentally adapted host-promiscuous serovars, while the newly evolved highly virulent clones, L2b and L3c, followed the trend of being unable to utilize the substrates (unpaired *t*-test) (Supplementary Fig. 3), in further support of host-restricted adaptation compensating for a loss of flexibility outside the host.

### Loss of fimbrial appendage switch to transovarial transmission and immune evasion

**Figure 5.**
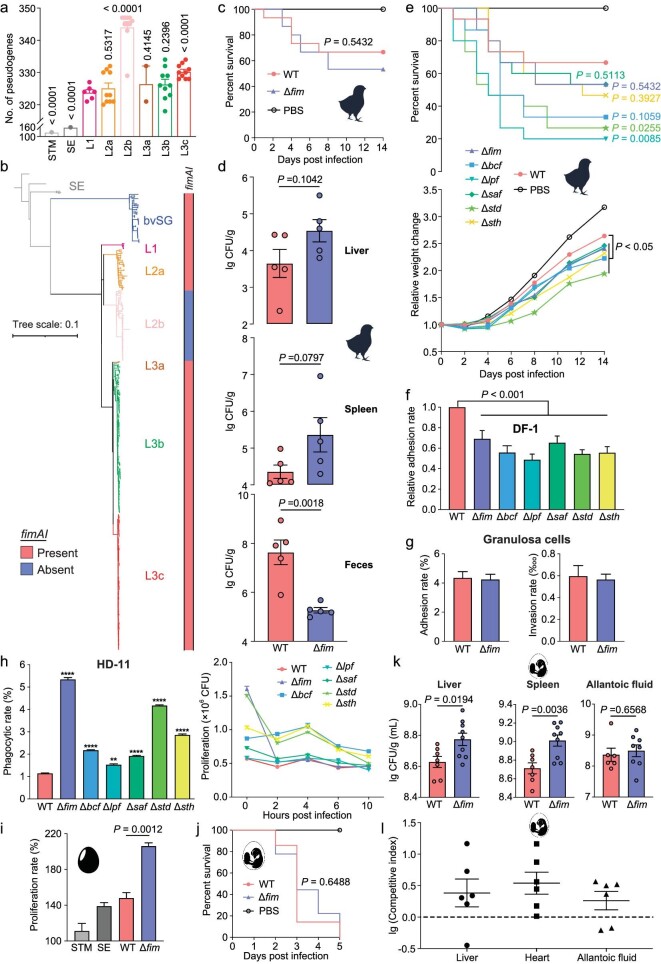
The impact of gene pseudolization on fimbrial appendages. (a) The number of pseudogenes among lineages. STM strain SL1344 and SE strain P125019 are used as controls. The *P* values between L1 and other lineages are shown above the columns. (b) Carriage of genes *fimA*-*fimI* among different lineages. (c) The survival curves of chicks (*n* = 15) infected with bvSP R51 wild-type and *fim* mutant strains. Chickens treated with PBS are used as controls. (d) The infected chick's liver, spleen and feces samples were harvested and tested for bacterial loads at 3 dpi (*n* = 5). (e) The survival curves and relative weight changes of chicks (*n* = 15) infected with bvSP R51 wild-type and fimbrial appendage mutant strains. Chicks treated with PBS are controls. (f) Bacterial adhesion assay for DF-1 cells. A comparison between R51 wild-type and fimbrial mutant strains was made. (g) Bacterial adhesion and invasion assay for granulosa cells. Comparisons were made between wild-type strain and *fim* mutant. (h) Bacterial phagocytosis and proliferation assay for HD-11 cells. A comparison was made between wild-type strain and fimbrial mutants. (i) The proliferation rate of a wild-type and *fim* mutant under egg albumen was compared. STM strain SL1344 and SE strain P125019 are used as controls. The proliferation rate was calculated as viable bacteria at 4 h/0 h × 100%. (j) The survival curves of chicken embryos (*n* = 7 to 9) infected with R51 wild-type and *fim* mutant. (k) The infected chicken embryo's liver, spleen and allantoic fluid samples were harvested and tested for bacterial loads at 5 dpi (*n* = 7 to 9). The allantoic fluid of two embryos was not available because they died near hatching. (l) The 16-day-old embryos were infected with a mix of wild-type and *fim* mutants for the competition assay. Competitive index values were calculated as the ratio of *fim* mutant to wild-type recovered from the liver, heart and allantoic fluid, divided by the ratio of two strains in the inoculum. The *P* values between groups of mutants and wild-type are shown on the right of the curves.

Host-restricted pathogens evolved from host-generalist pathogens with the hallmark of genome degradation and the acquisition of specific virulence genes [37–39]. To test the hypothesis, pan-genome analysis and pseudogene comparison were conducted (unpaired *t*-test) (Fig. 5a and Supplementary Fig. 4). We detected an enrichment of pseudogenes in sublineages with higher virulence, accompanied by host-adaptive evolution with increasing virulence. By conducting a pan-genome analysis, we found gene loss and acquisition among lineages. Notably, a deletion in the fimbrial gene cluster, particularly the genes (*fimA* and *fimI*) encoding the critical fimbrial subunit, was lost in L2b (Fig. 5b). Additionally, we observed a relatively high number of pseudogenes accumulated in L2b, which was correlated with higher virulence when compared with L2a (Fig. 3a–c).

To further investigate whether the loss of *fim* was associated with virulence and transmission behavior in L3, the type I fimbriae mutation of L3c strain R51 was used, in which *fimD* (usher, essential for fimbriae assembly on the surface of the cell) was deleted using the established CRISPR-Cas9 system. We found that the *fim* mutant promoted bvSP load in the liver (*P* = 0.1042; unpaired *t*-test) and spleen (*P* = 0.0797) in the chick infection model (Fig. 5c and d), indicating a virulence feature due to the loss of *fim.* The deletion of the other five fimbrial appendages in bvSP also confirmed the enhanced virulence behavior [40] (Fig. 5e and Supplementary Fig. 5). Notably, we found a significant decrease in the *fim* mutants in the feces (*P* = 0.0018) (Fig. 5d), suggesting a fitness defect in horizontal transmission (Supplementary Fig. 5c, d). This result was consistent with well-documented knowledge that fimbriae are responsible for avian host specificity and favor horizontal transmission [6,41–44]. Next, we assessed whether certain chicken epithelial cells (DF-1) and primary ovary cells (granulosa cells) play a role when interacting with fimbrial mutants, and we found a decrease in DF-1 cell adherence (Fig. 5f), indicating the role of decreased horizontal transmission ability in the mutants. No significant difference was observed in granulosa cells (Fig. 5g), indirectly suggesting that *fim* is not involved with interaction with ovary tissue. Collectively, the results suggest that fimbrial mutants could enhance virulence but may decrease horizontal transmission ability.

Considering the obligated pathogen, we then speculated that the role of macrophages is as a vehicle for bvSP dissemination. We assessed if fimbriae play a role in HD-11 macrophage interaction and observed that all fimbrial mutants could accelerate the uptake by the HD-11. This is particularly obvious in comparison to the *fim* mutant (Fig. 5h). Additionally, all fimbrial mutants could establish long-term survival in HD-11 macrophages (Fig. 5h), further suggesting that macrophages could be the key shelter for bvSP to achieve systematic dissemination, and fimbrial mutants, particularly *fim* mutants, have a significant advantage. Next, we use bacterial survival rate in the egg albumen (which contains various bactericidal substances to inhibit bacterial growth) and bacterial load in the chicken embryo (which considered as the vehicle for transovarial transmission) to evaluate the role of potential vertical transmission. The deletion of *fim* significantly promoted the bvSP proliferation in the egg albumen, suggesting a fitness advantage for vertical transmission (*P* = 0.0012; unpaired *t*-test). In contrast, the proliferation of host-promiscuous serovars was weak (Fig. 5i). For the *in vivo* chicken embryo infection model, loss of *fim* did not affect lethality (*P* = 0.6488; Log-rank Mantel-Cox test), but notably, it increased the bacterial load in the liver (*P* = 0.0194; unpaired *t*-test) and spleen (*P* = 0.0036) (Fig. 5j and k), further providing evidence that it may drive transovarial transmission. Further chicken embryo competition assays confirmed a fitness advantage for loss of *fim* (Fig. 5l), which again suggests that a loss of fimbrial appendages may result in a switch to transovarial transmission.

Finally, considering FimH as a TLR-4 ligand inducing a potent innate immune response [45,46], we speculated that such virulence or transmission behavior switches might be due to the immunological response modulated by the fimbrial mutant. The HD-11 cell infection assays demonstrated a generally reduced inflammation for most of the fimbrial mutants, in particular for chCXCLi1, iNOS, IL-8, TLR-4 and IFN-γ, which modulate inflammation for bacterial clearance [47] (Supplementary Fig. 6a). This was consistent with the fact that there are reduced replications within macrophage HD11 for recently evolved lineages (Fig. 3d) and fimbrial mutants (Fig. 5h), with less proinflammatory cytokine expression. Additional *in vivo* chick infection experiments also suggested a slight decrease in some proinflammatory cytokines, i.e. chCXCLi1, iNOS and IL-8 (Supplementary Fig. 6b). These differences may suggest that microbiota or other host factors may also play a role. Altogether, the fimbrial mutants in bvSP may result in persistent replication with reduced proinflammation in chicken macrophages, therefore changing the virulence and switching the transmission mode.

### Modern farming may accelerate bvSP virulence and transmission

**Figure 6.**
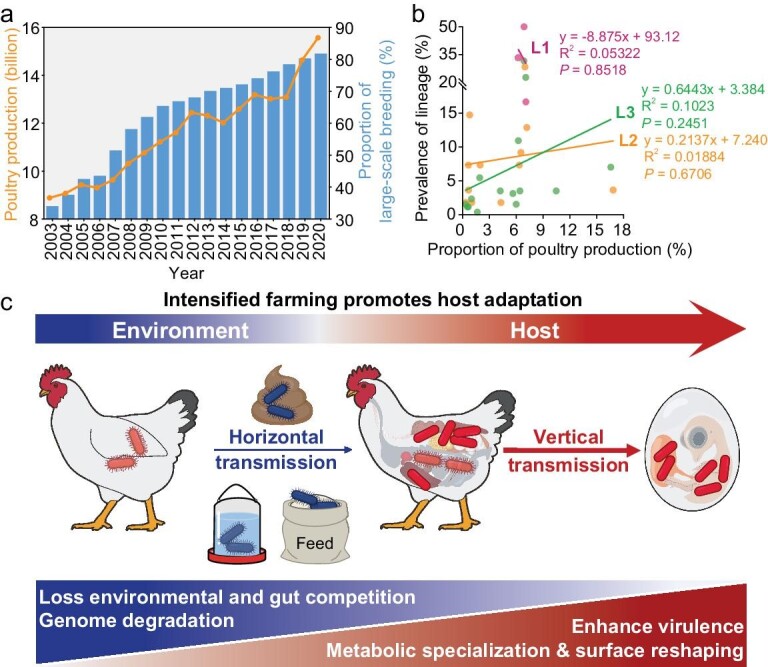
Intensified farming and the emergence of virulent clones. (a) The poultry farming style has dramatically changed in China. The annual stock of layers exceeds 2000, or the annual production of broilers exceeds 10 000 is defined as large-scale breeding. Data are from the National Bureau of Statistics (http://www.stats.gov.cn/). (b) Linear regression analysis with variables of lineage prevalence and poultry production (X-axis: total poultry production in the individual province between 1995 and 2020/total poultry production in China between 1995 and 2020 × 100%; Y-axis: distribution of bvSP lineages in China by provinces). Colored bubbles and lines refer to lineage. Data on poultry production are from the 2020 China Animal Husbandry and Veterinary Statistics. (c) The proposed host-adaptive evolution pathway for bvSP.

China is a leading poultry producer but has suffered economic losses as a result of bvSP [26] for decades, and its husbandry system has changed drastically in recent years (Fig. 6a and Supplementary Table 8). Interestingly, we found there might be a trend, in that the highly virulent lineages occurred preferentially in provinces with high poultry production (Fig. 6b). Therefore, we speculated that industrialized poultry farming might have accelerated the development of virulence in bvSP (Fig. 6c). Modern farming style, i.e. large-scale, intensified and layered cage-rearing of poultry, particularly for industrialized breeding, might provide an ideal niche for bvSP vertical transmission, in which the bacteria likely further spread via the transovarial pathway through egg-chick generations. Importantly, such a unique farming style may select variants to escape competition in the environment and host gut, driving host-restricted transmission but promoting the survival of invasive variants. To further reconfigure the increasing prevalence of high-risk bvSP clones in the poultry industry, we have established a multiplex Polymerase Chain Reaction (PCR) panel and demonstrated that it could efficiently differentiate the sublineages among clinical isolates (Supplementary Fig. 7 and Supplementary Table 9).

It is widely acknowledged that modern farming may provide a reservoir for pathogens with zoonotic potential, and this phenomenon has been previously correlated with the emergence of antimicrobial resistance [5,48,49]. A genomic investigation of the bvSP population provides a framework for understanding the diversity and biological consequences among lineages. A loss of environmental flexibility, as previously observed in the enrichment of pseudogenes in the metabolic pathway [11], is a hallmark of bvSP host-adaptive evolution. Importantly, a loss of H_2_S production could lead to a failure to compete with intestinal microbiota [50], and a unique mechanism of removing surface appendages could promote transovarial transmission, which coincides with the evidence that loss of flagella reduces intestinal inflammation and drives invasive infections [47,51,52]. The phenomenon of a shift of the outer membrane profiles for host-adaptive evolution was generally witnessed in other obligate pathogens, i.e. *Mycobacterium* [9] and *Yersinia* [53]. A stepwise loss of metabolic genes and surface appendages may change a pathogen from being versatile with predominant fecal–oral transmission to obligate with professional transovarial transmission.

## MATERIALS AND METHODS

### Bacterial isolates and chicken cells

A total of 509 genomes of *Salmonella enterica* serovar Gallinarum (*S.* Gallinarum) isolates collected between 1920 and 2022 were included, consisting of 458 bvSP, 49 bvSG and 2 bvSD. The whole genomic sequencing was conducted by Beijing Novogene Co. Ltd. A total of 305 isolates were stored in our laboratory and were identified by a one-step multiplex PCR assay, as described previously [54]. And sequences from 16 isolates were shared by our cooperators, the closed genome sequence of bvSP R51 isolate was conducted by Beijing Novogene Co. Ltd. Additionally, sequences from 188 isolates were obtained from public databases with whole genomic sequencing data (137 from Enterobase, 21 from Genbank, and 30 from Sequence Read Archive). Among the 458 bvSP isolates, 382 were from China and 76 were international isolates. The overall collection represented at least 14 countries, namely China (*n* = 385), Brazil (*n* = 25), UK (*n* = 24), USA (*n* = 13), Germany (*n* = 9), Denmark (*n* = 8), Nigeria (*n* = 7), Sweden (*n* = 6), Colombia (*n* = 4), France (*n* = 2), Mexico (*n* = 2), Belize (*n* = 1), Canada (*n* = 1) and India (*n* = 1), and 27 international isolates without an indicated country. *Salmonella* Typhimurium, Enteritidis, Newport, Dublin, Senftenberg, London and Indiana strains, and bvSP R51 were used routinely in the laboratory. All bacteria were cultured in Luria-Bertani broth at 37°C.

The granulosa cells were isolated from mature follicles (F1–F5) of 250-day-old Hy-line hens and cultured in Dulbecco's modified Eagle's medium (DMEM) high glucose (Hyclone, Tauranga, New Zealand) supplemented with 5% fetal bovine serum (Prime, EXCell Bio, China) [55].

The chicken macrophage-like cell line HD11 and chicken embryonic fibroblasts (DF-1) were maintained in DMEM (Gibco, USA) supplemented with 5% fetal bovine serum (Prime, EXCell Bio, China) at 37°C with 5% CO_2_.

### Antimicrobial susceptibility testing and antimicrobial resistance index

For 232 of the 305 *S.* Gallinarum from our laboratory, antimicrobial resistance was determined by a minimum inhibitory concentration (MIC) assay using the broth microdilution method according to the criteria recommended by the Clinical and Laboratory Standards Institute (CLSI-2016), as described previously [56]. *Escherichia coli* ATCC 25922 and *Pseudomonas aeruginosa* ATCC 27853 were used as quality control strains. The following 12 antimicrobial agents belonging to 8 classes were tested: aminoglycosides (gentamicin: GEN; kanamycin: KAN, streptomycin: STR), cephems (cefoxitin: FOX; ceftriaxone: CRO), penicillins (ampicillin: AMP), β-lactam combinations (amoxicillin-clavulanic acid: AMC), quinolones (ciprofloxacin: CIP; nalidixic acid: NAL), tetracyclines (tetracycline: TET), macrolides (azithromycin: AZM) and phenicols (chloramphenicol: CHL). Isolates with MICs in the intermediate range were classified as resistant for easy analysis. MDR refers to resistance to at least three antimicrobial classes. To facilitate comparison and visualization of antimicrobial resistance among isolates, the total antimicrobial resistance (Tar) value was introduced to quantify the overall antimicrobial resistance profile of an individual isolate using the following formula:


\begin{equation*}
Tar = \frac{1}{n}\mathop \sum \limits_{k = 1}^n \frac{{MIC}}{R}
\end{equation*}

where *n* is the number of antimicrobial agents, *MIC* is the minimum inhibitory concentration for each antimicrobial agent (mg/L) and *R* is the resistance breakpoint for each antimicrobial agent (mg/L).

### Ethical statements

The protocols of the animal studies were approved by the Committee of the Laboratory Animal Center of Zhejiang University (ZJU20190093; ZJU20190094; ZJU20220295).

## Supplementary Material

nwad228_Supplemental_Files

## Data Availability

Data availability is mentioned at the appropriate places within the manuscript.
