# Supplementary material for: Genome degradation promotes Salmonella pathoadaptation by remodeling fimbriae-mediated proinflammatory response
Source: Natl Sci Rev. 2023 Sep 2;10(10):nwad228. doi: 10.1093/nsr/nwad228 (PMC10642762; doi:10.1093/nsr/nwad228)
Supplement: nwad228_Supplemental_Files [file nwad228_supplemental_files.zip › Supplemetary data.docx]

**Genome degradation promotes *Salmonella* pathoadaptation by remodeling fimbriae-mediated proinflammatory response**

Xiao Zhou^1, 3 #^, Xiamei Kang^1 #^, Jiaqi Chen^1^, Yan Song^1^, Chenghao Jia^1, 2^, Lin Teng^1^, Yanting Tang^1^, Zhijie Jiang^1^, Xianqi Peng^1^, Xiaoxi Tao^1^, Yiwei Xu^1^, Linlin Huang^1^, Xuebin Xu^4^, Yaohui Xu^5^, Tengfei Zhang^6^, Shenye Yu^7^, Jiansen Gong^8^, Shaohui Wang^9^, Yuqing Liu^10^, Guoqiang Zhu^11^, Corinna Kehrenberg^12^, François-Xavier Weill^13^, Paul Barrow^14^, Yan Li^1, 2^, Guoping Zhao^15, 16, 17^, Min Yue^1, 2, 18, 19 *^

^1^ Institute of Preventive Veterinary Sciences & Department of Veterinary Medicine, Zhejiang University College of Animal Sciences, Hangzhou, 310058, China

^2^ Hainan Institute of Zhejiang University, Sanya 572025, China

^3^ Ningbo Academy of Agricultural Sciences, Ningbo 315040, China

^4^ Department of Microbiology Laboratory, Shanghai Municipal Center for Disease Control and Prevention, Shanghai 200336, China

^5^ College of Veterinary Medicine, Henan University of Animal Husbandry and Economy, Zhengzhou 450053, China

^6^ Key Laboratory of Prevention and Control Agents for Animal Bacteriosis, Institute of Animal Husbandry and Veterinary, Hubei Academy of Agricultural Sciences, Wuhan 430064, China

^7^ Division of Bacterial Diseases, State Key Laboratory of Veterinary Biotechnology, Harbin Veterinary Research Institute, Chinese Academy of Agricultural Sciences, Harbin 150069, China

^8^ Poultry Institute, Chinese Academy of Agricultural Sciences, Yangzhou 225125, China

^9^ Department of Animal Public Health, Shanghai Veterinary Research Institute, Chinese Academy of Agricultural Sciences, Shanghai 200241, China

^10^ Shandong Key Laboratory of Animal Disease Control and Breeding, Institute of Animal Science and Veterinary Medicine, Shandong Academy of Agricultural Sciences, Jinan 250100, China

^11^ College of Veterinary Medicine, Yangzhou University, Yangzhou 225009, China

^12^ Institute for Veterinary Food Science, Faculty of Veterinary Medicine, Justus-Liebig University Giessen, Giessen 35392, Germany

^13^ Institut Pasteur, Université Paris Cité, Unité des bactéries pathogènes entériques, 75724 Paris cedex 15, Paris, France

^14^ School of Veterinary Medicine, University of Surrey, Daphne Jackson Road, Guildford GU2 7AL, United Kingdom

^15^ School of Life Science, Hangzhou Institute for Advanced Study, University of Chinese Academy of Sciences, Hangzhou, 310024, China.

^16^ CAS Key Laboratory of Synthetic Biology, Institute of Plant Physiology and Ecology, Shanghai Institutes for Biological Sciences, Chinese Academy of Sciences, Shanghai 200031, China.

^17^ Department of Microbiology and Microbial Engineering, School of Life Sciences, Fudan University, Shanghai 200433, China.

^18^ State Key Laboratory for Diagnosis and Treatment of Infectious Diseases, National Clinical Research Center for Infectious Diseases, National Medical Center for Infectious Diseases, The First Affiliated Hospital, College of Medicine, Zhejiang University, Hangzhou 310003, China

^19^ Zhejiang Provincial Key Laboratory of Preventive Veterinary Medicine, Hangzhou 310058, China

# XZ and XK contributed equally to this work.

* Correspondence: Min Yue ([myue@zju.edu.cn](mailto:myue@zju.edu.cn))

**SUPPLEMENTARY MATERIALS AND METHODS**

***Short-read sequencing technology***

A total of 305 *S*. Gallinarum, including 299 bvSP, in our laboratory were subjected to whole-genome sequencing. Genomic DNA extraction of overnight cultures was performed using the FastPure Bacteria DNA Isolation Mini Kit (Vazyme Biotech Co., Ltd.) according to the manufacturer's recommendations. The harvested genomic DNA was detected by agarose gel electrophoresis and quantified by NanoDrop1000 (Thermo Fisher Scientific, USA). The libraries were constructed and sequenced on an Illumina Novaseq 6000 platform in 150 bp paired-end mode (Novogene, Beijing, China). Sequence data were deposited at the NCBI Sequencing Read Archive (SRA) under BioProject accession PRJNA850899.

***Genomic data analysis***

The quality of all raw reads, including raw data from *S*. Gallinarum isolates in our laboratory (n = 321) and public databases (n = 188), was evaluated by FastQC v.0.11.9 (<https://www.bioinformatics.babraham.ac.uk/projects/fastqc/>) and low-quality and adapter sequences were removed by Trimmomatic v.0.38.1 [1]. *De novo* assembly was performed by SPAdes v.3.12.0 to generate contigs [2]. All the subjected isolates were confirmed as *S*. Gallinarum by *in silico* serotyping conducted by SISTR v.1.1.1 [3]. Subsequently, multilocus sequence typing (MLST) was performed by MLST v.2.19.0 (<https://github.com/tseemann/mlst>). Antimicrobial resistance genes (ARGs), plasmids, and virulence factors were analysed by ResFinder v.4.0, PlasmidFinder v.2.1.1, and VFDB on ABRicate v.1.0.1, respectively (<http://www.github.com/tseemann/abricate>). *De novo* plasmid assembly was performed by plasmidSPAdes v.3.9 to reconstruct plasmids [4], on which ARGs were screened by Resistance Gene Identifier v.5 to predict the location of ARGs (on chromosome or plasmid) [5].

***Long-read sequencing, assembly, and annotation***

The complete genome of the highly virulent bvSP strain R51 recovered from a chicken liver was sequenced. Genomic DNA was extracted with the SDS method, as described previously [6]. The quality and quantity of genomic DNA were examined by agarose gel electrophoresis and a Qubit® 2.0 Fluorometer (Thermo Fisher Scientific, USA). Libraries for single-molecule real-time (SMRT) sequencing were generated with an insert size of 10 kb using the SMRT bell™ Template kit v.1.0 (Pacific Biosciences of California, USA), while libraries for Illumina sequencing were constructed using NEBNext® UltraTM™ DNA Library Prep Kit (NEB, USA) following the manufacturer’s instructions. The whole genome of *Salmonella* Pullorum R51 was sequenced by the PacBio Sequel platform and Illumina NovaSeq PE150 at the Beijing Novogene Bioinformatics Technology Co., Ltd.

The clean data was obtained by filtering low-quality reads (less than 500 bp) to ensure the accuracy of the results of the subsequent analysis. Sequences were assembled and corrected by SMRT Link v.5.0.1. The assembly result was further checked with Illumina data to realize the cyclization and initiation site corrections.

Genome annotation was conducted using NCBI Prokaryotic Genome Annotation Pipeline (PGAP) v.5.0 (https://www.ncbi.nlm.nih.gov/genome/annotation_prok/). The genome was submitted to the NCBI Genome under BioProject accession PRJNA689917.

***Phylogenetic analysis***

The complete genome of bvSP R51 presented in this study was used as a reference for core-genome single nucleotide polymorphism (SNP) calling. A total of 17518 SNPs in the core genome were identified by Snippy v.4.4.5 (https://github.com/tseemann/snippy). The recombination regions were filtered from the core genome alignment by Gubbins v.2.3.4 [7]. The maximum-likelihood phylogenetic tree was generated using the best model (TVM+F) by IQ-TREE v.1.6.12 [8]. *Salmonella* Enteritidis P125109 (accession no. GCA_015240635.1) was used as an outgroup to root the tree. The annotation and visualization of the final phylogenetic tree was projected on interactive Tree Of Life (iTOL) v.5 [9] (https://itol.embl.de).

***Chicken embryo infection assay***

The virulence of 31 *S*. Gallinarum strains from different lineages was assessed in 16-day-old SPF chicken embryos using inoculation of the allantoic cavity. Uninoculated embryos and inoculation with phosphate-buffered saline (PBS) were used as controls. Chicken embryos were incubated in a thermostatic incubator at 37.5°C with a relative humidity of 50–60%. The viability of the embryos was checked according to the integrity of the venous system and the movement of the embryos before inoculation, and dead embryos were discarded. 1000 CFU in 100 μL of overnight cultures resuspended in PBS were administered. Deaths were recorded daily for five days until the day of hatching.

The role of type I fimbriae in virulence evolution was investigated using the chicken embryo model described above with minor modifications. Briefly, wild-type and mutant strains of Pullorum R51 were administered with an optimal infectious dose (200 CFU in 100 μL, n= 7 or 9), which was determined by pilot experiments. The survival of the embryos was checked every day post-inoculation. On day five, all the embryos were dissected aseptically for the determination of the bacterial burden in the liver, spleen, and allantoic fluid, respectively. Tissue samples were weighed and homogenized with zirconia beads in PBS using a Tissuelyser-24 (Jingxin, Shanghai, China), and the homogenates were 10-fold-serially diluted and spread onto LB agar to quantify the bacterial loads.

### *Chick embryo competition assay*

For the competition assay, 16-day-old embryos were inoculated via the allantoic cavity with a mix of equal numbers of wild-type R51, which had been introduced with a plasmid carrying the kanamycin resistance gene, and *fim* mutant (total 200 CFU in 100 μL of PBS). The numbers of two strains in the inoculum and in the liver, heart and allantoic fluid of embryos on the fifth day after infection were calculated by plating onto LB containing 200 μg/mL nalidixic acid (for wild-type and *fim* mutant) or 50 μg/mL kanamycin (only for wild-type). The competitive index was calculated as the ratio between the *fim* mutant and wild-type strain, divided by the same ratio in the inoculum.

***Chick infection assay***

Six *S*. Gallinarum strains were selected for the chick infection model: bvSP strain SAL03394 (L1), MY0188 (L2a), MY0334 (L2b), MY0189 (L3b), and SAL00744 (L3c), and bvSG strain MY0187. Fertilized SPF chicken embryos were purchased from a commercial hatchery and hatched in a sterile environment in our laboratory. The virulence of six strains was evaluated in one-day-old chicks by oral administration. Cloacal swabs were collected from all chicks before the infection to ensure they were *Salmonella* free. Chicks were divided randomly into seven groups (bvSG: n = 9; bvSP: at least 30 chicks per group) and infected with each strain (10^9^ CFU in 200μL) and 200μL PBS as control, respectively. Deaths were observed and recorded daily for 14 days, and clinical symptoms of each chick were documented individually based on the scoring system: 0, normal; 1, growth retardation; 2, white diarrhoea; 3, skelasthenia; 4, depression or dyspnea; 5, dying. Five randomly selected chicks from each group were euthanized and dissected on day seven post-infection. The bacterial load of tissues, including the heart, liver, and spleen, was determined as described in the chicken embryo infection models.

The role of six fimbrial appendages in virulence evolution was investigated by the chick infection model described above with minor modifications. Briefly, chicks were randomly divided into eight groups (n = 48 to 51, 15 chicks labeled for survival observation, and 33 to 36 chicks for sampling in each group) and oral administration of each strain (10^7^ CFU in 200μL) and 200μL PBS, respectively. Deaths were recorded daily for 14 days. Weights were recorded every two days for 14 days. Five chicks selected randomly from each wild-type and the mutant group were euthanized and dissected on day three post-infection to determine the bacterial load of tissues, including the liver, spleen, and feces, as described in the chicken embryo infection models. And tissues, including the liver, spleen, and cecum, were collected to evaluate pathological changes on day five post-infection.

In order to investigate whether the *fim* mutant affects the host's immune response, 10^7^ CFU wild-type and *fim* mutant were orally administered to one-day-old chicks, PBS serving as the control. On day three post-infection, liver, spleen, and cecum samples were collected for tissue RNA extraction.

***HD11 phagocytosis and proliferation assay***

Five bvSP strains were used in the cell model: SAL03394 (L1), MY0188 (L2a), MY0334 (L2b), MY0189 (L3b), and SAL00744 (L3c). *Salmonella* Typhimurium strain SL1344 and *Salmonella* Enteritidis strain P125109 were used as controls.

Cells (2 × 10^5^ cells per well) were grown for 12 h in 12-well plates and infected with selected strains with a multiplicity of infection (MOI) of 50:1 for 1 h. Subsequently, the cells were gently washed three times with PBS and treated with 100 μg/mL gentamicin in the medium for 1 h to kill extracellular bacteria. The cells were washed twice with PBS and lysed with 1 ml 0.1% Triton X-100. The lysates were 10-fold serially diluted and plated onto LB agar to quantify the intracellular bacteria. The phagocytosis rate (%) was calculated as (intracellular bacteria / infection bacteria) × 100%. The infected cells were cultured in the medium containing 10 μg/mL gentamicin. Cells were washed and lysed at 12 hpi to determine the intracellular bacteria as described above. The proliferation rate was calculated as intracellular bacteria at 12 hpi / intracellular bacteria at 1 hpi. Three independent experiments were performed in triplicate for each strain.

The wild-type and six fimbrial appendage mutants (Δ*fim,* Δ*bcf,* Δ*lpf,* Δ*saf,* Δ*std,* Δ*sth*) were used to infect HD11 cells as described above. The time point of phagocytosis served as the starting point for proliferation, and the number of bacteria in cells was counted after 2, 4, 6 and 10 hours of proliferation.

***DF-1 cell adhesion assay***

The adhesion of R51 wild-type and fimbrial appendage mutant strains to DF-1 cells was tested. Cells (4 × 10^5^ cells/mL) were grown for 12 h in 12-well plates and infected with bacteria with an MOI of 50:1 for 1 h. Subsequently, the cells were washed gently three times with PBS and lysed with 1 ml 0.1% Triton X-100. The lysates were 10-fold serially diluted and plated onto LB agar to quantify the bacteria adhering to the cells. The adhesion rate (%) was calculated as (bacteria adhering to the cells / infection bacteria) × 100%. The relative adhesion rate was calculated as (the adhesion rate of mutant strains / the adhesion rate of wild-type R51). Three independent experiments were performed in triplicate for each strain.

***Granulosa cells adhesion and invasion assay***

The adhesion and invasion abilities of WT and *fim* mutant in granulosa cells were investigated. The granulosa cells were grown in 24-well plates (2.5×10^5^ cells/ well) and infected with bacteria with an MOI of 50:1 for 1 hour. The calculation of adhesion rate was the same as for DF-1 cell mentioned above. Meanwhile, the remaining plates were further incubated in the medium containing 10 μg/mL gentamicin for 1 hour. The invasion rate was calculated as (intracellular bacteria / infection bacteria) × 100%.

***Calculation of invasiveness index***

The invasiveness index was calculated as described previously [10,11]. Briefly, we used the random forest model trained by the former method together with R v.4.1.2 to calculate the invasiveness index. This model showed the applicability of calculating the invasiveness index for different species of *Salmonella*. Specifically, the DeltaBS metric was used to identify mutations in protein-coding genes in the Whole Genome Sequence of the strains used for model building, from which 196 top predicted genes were obtained for measuring invasiveness in *S. enterica*. The invasiveness index calculation code used is available at https://github.com/Gardner-BinfLab/invasive_salmonella.

***Desiccation, acid and alkali stress assays***

Four isolates of each sublineage (only three isolates available in L1: MY0186, SAL03394, SAL03395; L2a: MY0188, MY0332, MY0335, SAL03405; L2b: MY0334, SAL02650, SAL03507, SAL04026; no available L3a isolates; L3b: MY0189, SAL02697, SAL03287, SAL04023; L3c: SAL00744, SAL02657, SAL03385, SAL03401) were selected to evaluate their resistance to desiccation, acid and alkali stress conditions in three biological replicates. *Salmonella* Typhimurium strain SL1344 and *Salmonella* Enteritidis strain P125109 were used as controls.

For the desiccation assay, overnight cultures were harvested, washed, resuspended, and adjusted to OD_600nm_ of 1 with 10 mM PBS (pH = 7.4). 50 μL of the suspensions were added to polystyrene 96-well microtiter plates and incubated at 22°C for 24 h in a sealed vessel containing saturated potassium acetate solution to maintain a relative humidity of 36%. The initial and final bacteria were quantified by culturing 10-fold serial dilutions on LB agar. The survival rate (%) was calculated (viable counts after treatment / initial counts) × 100%.

For acid and alkali stress assays, normal saline was adjusted to pH = 2 and 11 with HCl and NaOH, respectively. The overnight cultures were harvested, washed, resuspended, and adjusted to OD_600nm_ of 0.5 with 10 mM PBS (pH = 7.4). 30 μL of the bacterial suspension was added into 270 μL normal saline with these other two different pH values, mixed and incubated at room temperature for 15 min and 10 min, respectively. The initial and final bacteria were quantified by culturing 10-fold serial dilutions on LB agar. The survival rate (%) was calculated (viable counts after treatment / initial counts) × 100%.

***Biofilm formation assay***

The biofilm formation assay of 228 strains was performed as described previously [12,13], with modifications. *Escherichia coli* ATCC 25922 was used as a control strain. Overnight cultures were adjusted to OD_600nm_ of 0.5 and diluted 1:100 in Tryptic Soy Broth (TSB). The diluted cultures were transferred to 96-well flat-bottom plates at 200 μL per well. To prevent evaporation, the experimental wells were set in the center of the 96-well plate, and 300 μL of double-distilled water was added to the peripheral wells, and were statically incubated at 28°C for 48 h. After incubation, the planktonic cells were discarded, and the wells were gently washed three times with double-distilled water, while the adherent cells were stained with 200 µL of 0.4% crystal violet for 15 min at room temperature. The wells were washed five times with double-distilled water to thoroughly remove crystal violet, not specifically staining the adherent bacteria. The remaining crystal violet bound to the adherent cells was dissolved with 200 μL of eluent (25% acetone with anhydrous ethanol) for 20 min. Biofilm was quantified by OD550_nm_ measured with a microplate reader (BioTek, USA). Three independent experiments were conducted in quadruplicate for each strain. *Salmonella* Typhimurium (n = 2) and *Salmonella* Enteritidis (n = 3) isolates were used as controls.

***Biochemical assays***

16 biochemical tests were performed and interpreted per individual bacterial strain under aerobic and anaerobic conditions, following the manufacturer’s instructions (Beijing Land Bridge Technology Co., Ltd). The following assays were included: fermentation of L-arabinose, mannose, L-rhamnose, D-glucose, D-xylose, tartrate, maltose, dulcitol, D-sorbitol and trehalose, ornithine decarboxylase activity, D-glucose gas production, arginine dihydrolase activity, citrate utilization and mucate, and H_2_S production. Briefly, a single colony was selected from freshly cultured plates of each strain to prepare a suspension of 0.5 McFarland in 2 mL of sterile water. The bacterial suspension obtained was dropped into the ampoule (three drops per bottle) and incubated at 37°C for 24 to 48 h under aerobic and anaerobic conditions. Three independent experiments were performed in triplicate for each strain.

***Pan-genome, pseudogene and comparative genomics analysis***

Genomes were annotated with reference genome R51 and gathered for pan-genome analysis using Prokka v.1.13 [14]. Large-scale genomic data annotation and the pan-genome gene family presence/absence matrix analysis were conducted by using Roary v.3.12.0 [15]. Further, the generated presence/absence matrix was reordered manually by lineages and visualized by iTOL v.5.

The number of pseudogenes was obtained manually from genome annotation data performed by NCBI Prokaryotic Genome Annotation Pipeline (PGAP) under BioProject accession PRJNA852692 and PRJNA853766.

Usher is the largest and most conserved protein of a fimbrial apparatus. Thus, the sequence of usher protein of *fim* gene cluster was used to search bvSP genomes (Genbank accession no. GCA_000235545.1, GCA_000330485.2, GCA_000444445.1, GCA_000462995.1, GCA_000484295.1, GCA_000486745.1, GCA_000521885.1, GCA_000953595.1, GCA_001448575.1, GCA_003606225.1, GCA_015024675.1, GCA_015024685.1, GCA_015024695.1, GCA_015024735.1) with tblastn (https://blast.ncbi.nlm.nih.gov/Blast.cgi?) to identify and collect all predicted genes and encoded proteins of fimbrial gene clusters based on our previous study [16].

***Construction of mutant strains***

The traceless construction of bvSP R51 fimbrial appendage mutant strains (Δ*fim,* Δ*bcf,* Δ*lpf,* Δ*saf,* Δ*std,* Δ*sth*) was performed by the two-plasmid-based CRISPR-Cas9 system, as described previously, with minor modifications [17–19]. Briefly, the sgRNA was predicted by CHOPCHOP (<http://chopchop.cbu.uib.no>) and amplified with primers sgRNA-F/sgRNA-R. The upstream and downstream fragments (500 bp) of the target gene were amplified with two pairs of primers (Xf-F/Xf-R and Xr-F/Xr-R) and fused to obtain donor DNA (1000 bp). The sgRNA and donor DNA fragments were purified by SanPrep Column PCR Product Purification Kit (Sangon Biotech (Shanghai) Co., Ltd.) and homologous recombined into plasmid pTargetF by Hieff Clone^®^ Plus One Step Cloning Kit (Yeasen Biotechnology (Shanghai) Co., Ltd.) to obtain plasmid pTargetT, which was identified by PCR with primers pTargetT-F/pTargetT-R and further sequenced by Zhejiang SunYa Biotech. Plasmid pCas and pTargetT were extracted by Easy Plasmid Miniprep Kit (Zhejiang Easy-Do Biotech Co., Ltd.) and chemically transformed into R51 competent cells to generate fimbrial appendage deletion mutant strains. To improve gene editing efficiency, L-arabinose (10 mM) was added to induce the expression of homologous recombinases on plasmid pCas. The gene deletion mutants were identified by colony PCR using 2×T5 Super PCR Mix (Colony, Tsingke Biotechnology Co., Ltd.) with primers Check-F/Check-R, detected by agarose gel electrophoresis (*Trans*2K^®^ DNA Marker, TransGen Biotech Co., Ltd), and further sequenced. Finally, plasmid pTargetT was cured by CRISPR-Cas9 system induced by isopro-pyl-D-thiogalactopyranoside (IPTG, 0.5 mM) and the temperature-sensitive plasmid pCas was cured by incubation at 42°C for three days. Primers for mutant construction are in **Supplementary Table 10**.

***Egg albumen tolerance assay***

The viability of wild-type and mutant strains in egg albumen was evaluated as described previously with minor modifications [20]. *Salmonella* Typhimurium strain SL1344 and *Salmonella* Enteritidis strain P125109 were used as controls. Briefly, fresh, organic, unfertilized, antibiotic-free eggs were disinfected with 70% ethanol. Egg albumen was collected aseptically, pooled, and determined to be sterile prior to the assay. The overnight cultures were diluted with sterile normal saline. 100 μL of the diluted cultures were added to 900 μL of egg albumen to a final concentration of 2-4 × 10^3^ CFU/mL and mixed thoroughly. After incubating at 37°C for 4 h and 24 h, the bacterium-albumen mixture was diluted with sterile normal saline and spread onto LB agar to enumerate the viable bacteria counts. Uninfected egg albumen was used as the negative control. The proliferation rate was calculated as viable bacteria after 4 h / viable bacteria after 0 h × 100%. Five independent experiments were conducted in triplicate for each strain.

***One-step multiplex PCR for differentiation sublineages***

A simple one-step multiplex PCR method was established to identify different sublineages among bvSP. And genomes of seven other serovars were included to validate the specificity of the method. We designed primers for specific genes obtained based on the pan-genome analysis of *S.* Gallinarum genomes **(Supplementary Table 11)**. Primers for gene *bcfC* and *rhs* were used to identify *S*. *enterica* and *S*. Gallinarum, respectively, as described previously with modifications [21]. Initially, monoplex PCR was performed to ensure the distribution and size of target genes **(Supplementary Table 12, 13)**. Briefly, the multiplex PCR was performed with 1 μL of DNA sample or overnight bacterial culture, 15 μL of KOD One PCR Master Mix (TOYOBO (Shanghai) Biotech Co., Ltd.), 10 µM of each primer (0.25 μL of primer *bcfC-*F/R, 0.68 μL of primer *rhs*-F/R, 0.28 μL of primer *pxpA*-F/R, 0.3 μL of primer *cycA*-F/R, 0.18 μL of primer group6154-F/R, 0.4 μL of primer *fimAI*-F/R, 0.18 μL of primer group3248-F/R, and 0.2 μL of primer group3898-F/R), and 9.06 μL ddH_2_O in a final volume of 30 µL. The PCR amplification was performed under the following reaction conditions: initial denaturation at 95°C for 10 min; 30 sequential cycles of denaturation at 98°C for 10 s, annealing at 57°C for 30 s, and extension at 72°C for 5 s; ending with a final extension at 72°C for 10 min. The PCR products were subjected to electrophoresis in 1% (w/v) agarose gel.

One isolate of each sublineage was selected to evaluate the limit of detection (LOD) of the multiplex PCR method: SAL03394 (L1), MY0188 (L2a), MY0334 (L2b), MY0189 (L3b), SAL00744 (L3c), and MY0187 (bvSG).

***Real-time quantitative PCR analysis***

Total RNA was extracted from HD11 cells and chick tissues using Total RNA Isolation Kit (BioTeke Corporation, Beijing, China). The RNA yield was determined by NanoDrop1000 (Thermo Fisher Scientific, USA). First-strand cDNA was synthesized from total RNA using HiScript II 1st Strand cDNA Synthesis Kit (+gDNA wiper) (Vazyme Biotech Co., Ltd.). Transcriptional levels of the target genes were analyzed using ChamQ Universal SYBR qPCR Master Mix (Vazyme Biotech Co., Ltd.) with an Agilent Mx3005P qPCR System (Agilent Technologies, USA). Relative gene expression was analyzed using the 2^-ΔΔ^*^CT^* method, and GAPDH was used as an internal control. The primers designed for qPCR are listed in **Supplementary Table 14**.

***Statistical analysis***

The results were projected and statistically analyzed by GraphPad Prism version 9 (San Diego, CA, USA). The unpaired *t*-test or ordinary one-way ANOVA was used to analyze the differences between isolates. Log-rank Mantel-Cox test was used to perform the comparison of the survival curve. Linear regression was used to analyze the temporal AMR trends and poultry production-virulence trends. The value was shown as the mean (SEM). *P* < 0.05 was considered statistically significant.

**SUPPLEMENTARY FIGURES**

**
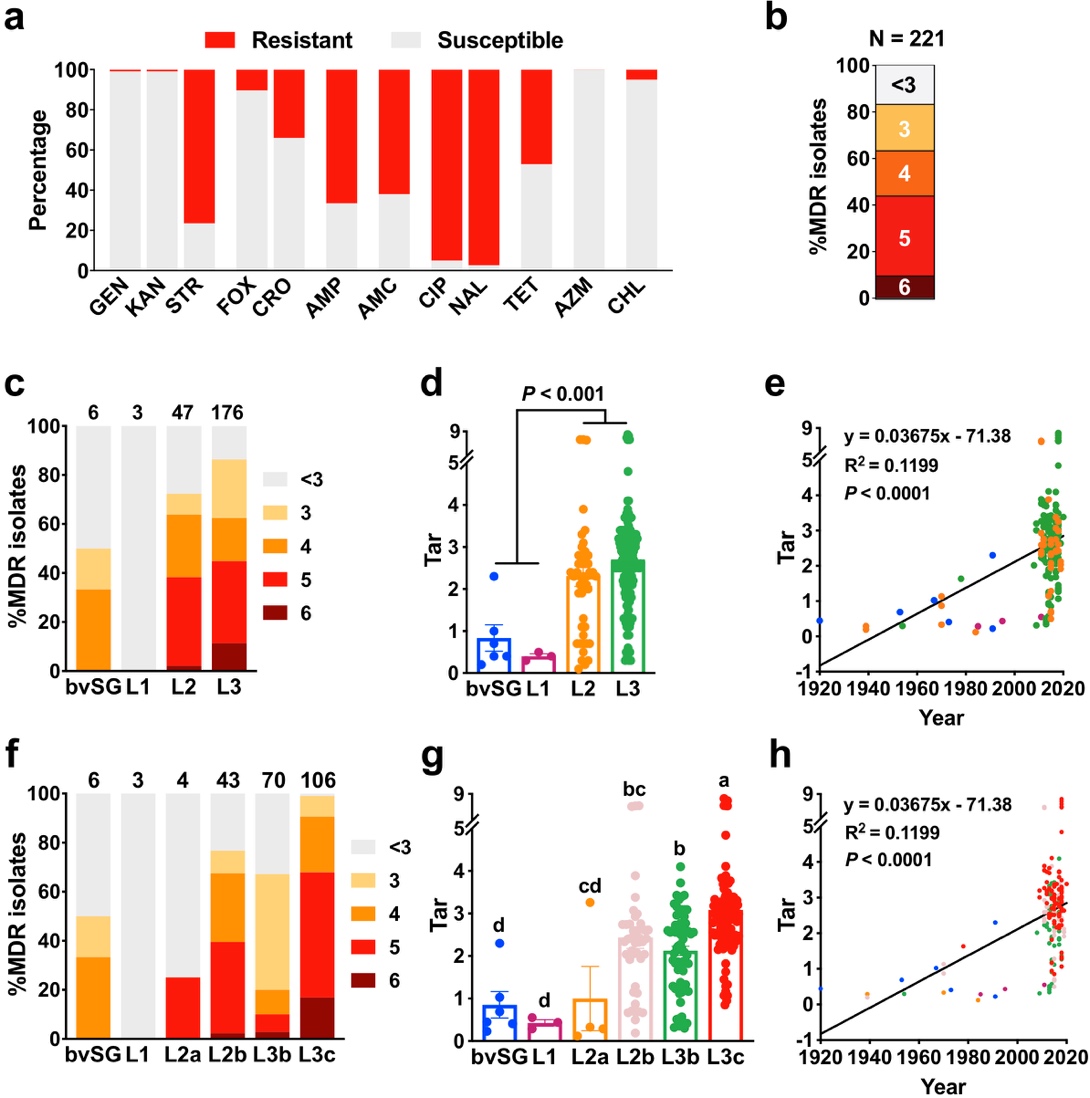
**

**Supplementary Figure 1. Distribution of antimicrobial resistance among *Salmonella* Pullorum isolates. (a)** Prevalence of antimicrobial resistance among the 221 *Salmonella* Pullorum isolates against 12 antimicrobial agents of eight classes. Additional gaps separate different classes of antimicrobial agents. The names of the antimicrobials are abbreviated as gentamicin (GEN), kanamycin (KAN), streptomycin (STR), cefoxitin (FOX), ceftriaxone (CRO), ampicillin (AMP), amoxicillin-clavulanic acid (AMC), ciprofloxacin (CIP), nalidixic acid (NAL), tetracycline (TET), azithromycin (AZM), and chloramphenicol (CHL). **(b)** The proportion of MDR isolates, with the number of antimicrobial classes shown in each block. **(c-h)** Dynamics of AMR in *Salmonella* Pullorum lineages. A total of 232 global isolates, including isolates of bvSG as a control, were examined against 12 antimicrobial agents of eight classes. To facilitate comparison and visualization, we propose a new index called total antimicrobial resistance (Tar) to represent overall AMR profiles for each bacterial isolate. **(c, f)** The distribution of MDR isolates. The color indicates the number of antimicrobial classes. The number of isolates in each lineage **(c)** and sublineage **(f)** is shown above the column. **(d, g)** Comparison of antimicrobial resistance among bvSP lineages **(d)** and sublineages **(g)**. Letters a, b, c, and d indicate statistically significant differences of *P* < 0.05 between sublineages. Bars with no common letters are significantly different (*P* < 0.05). **(e, h)** The time-scale trend for AMR profiles among different lineages **(e)** and sublineages **(h)**. Linear regression analysis with variables of Tar value and time. The colored dots refer to individual lineages or sublineages.


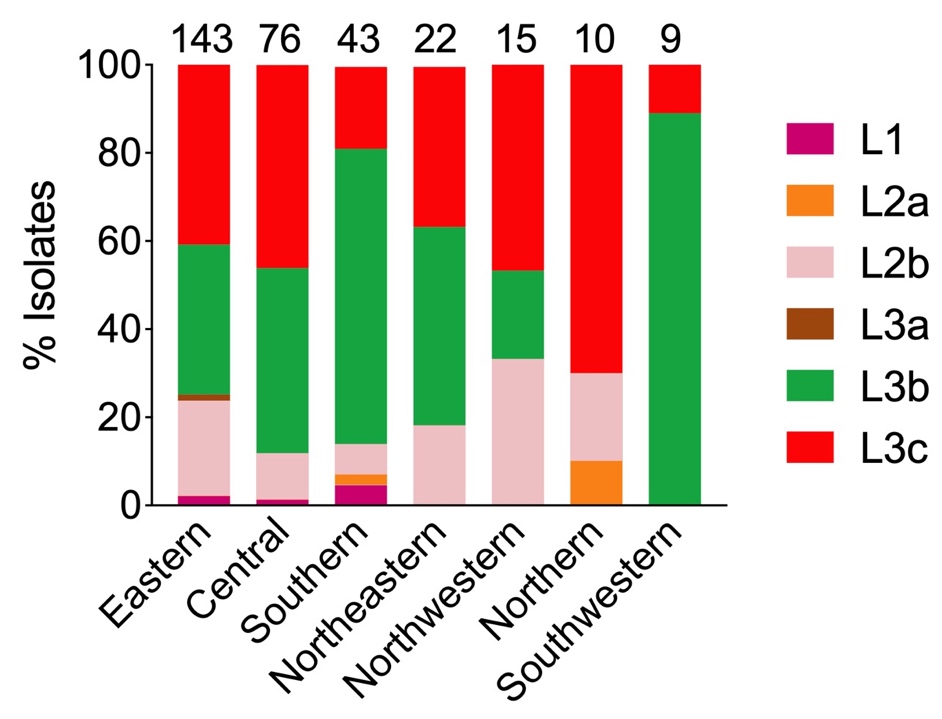


**Supplementary Figure 2. Lineage diversity of *Salmonella* Pullorum in China.** The proportion of lineages collected in the corresponding region, with the total number of isolates shown above each bar. Two strains of unknown geographic origin are not shown.


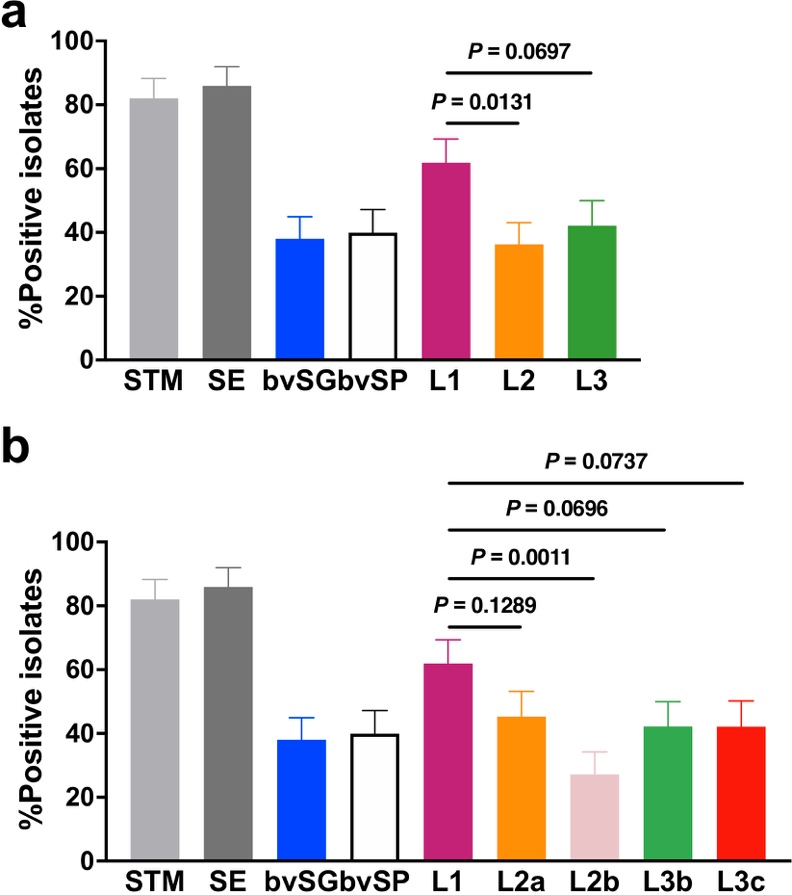


**Supplementary Figure 3. Trends in the biochemical metabolic capacity of *Salmonella* Pullorum lineages.** The column height refers to the arithmetic average of the utilization of 16 compounds under aerobic and anaerobic conditions for each lineage. *Salmonella* Typhimurium, *Salmonella* Enteritidis and bvSG are control species. **(a)** compares lineages. **(b)** compares sublineages.


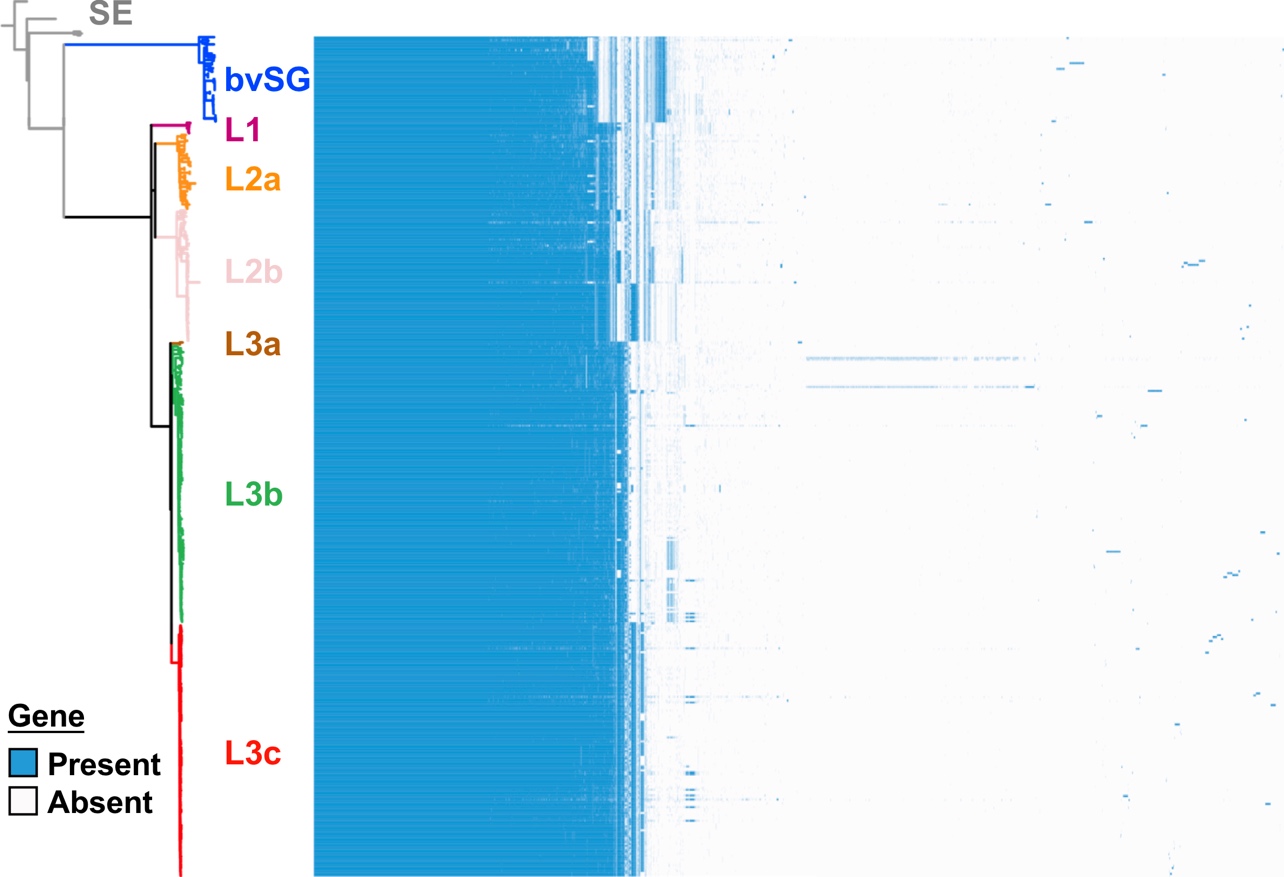


**Supplementary Figure 4. Pan-genome analysis of *Salmonella* Pullorum isolates.** The pan-genome is visualized as a heatmap showing the presence (blue) and absence (light grey) of genes and is ordered from the core genes on the left to the accessory genes on the right. Genomes are sorted by lineages assigned with different colors.


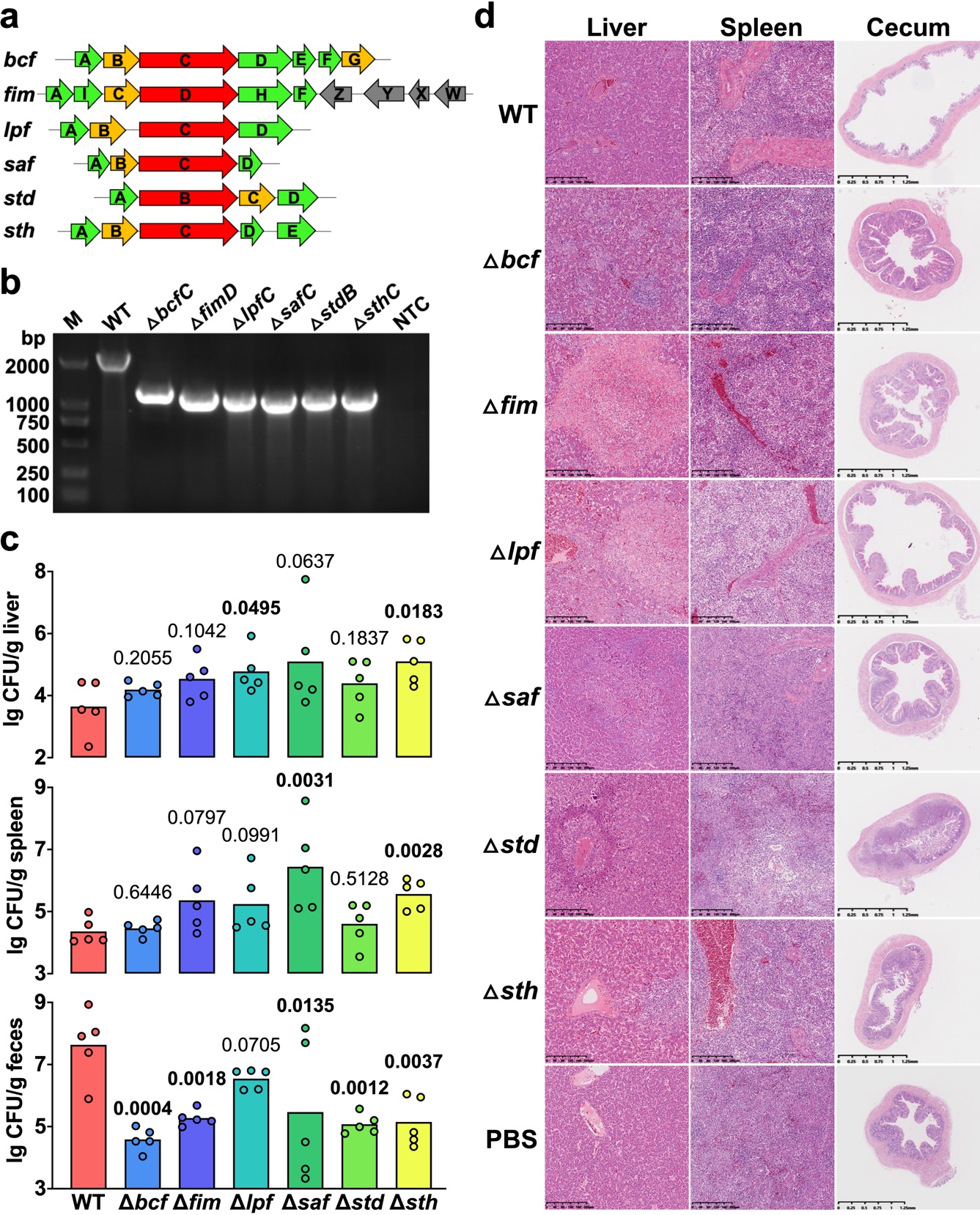


**Supplementary Figure 5. Deletion of fimbriae alters the niche of bvSP within the host. (a)** Identification of chaperone-usher fimbrial gene clusters of bvSP based on comparative genomics analysis. Colored arrows show type: subunit, green; chaperone, orange; usher, red; regulator, grey. **(b)** Confirmation of fimbrial usher deletion by PCR. M: DNA marker; NTC: negative control. **(c)** The infected chick’s liver, spleen, and feces samples were harvested and tested for bacterial loads at 3 dpi (n = 5). The *P* values between groups of mutants and wild-type are shown above the columns. **(d)** The infected chick’s liver, spleen, and cecum samples were collected to evaluate pathological changes at 5 dpi.


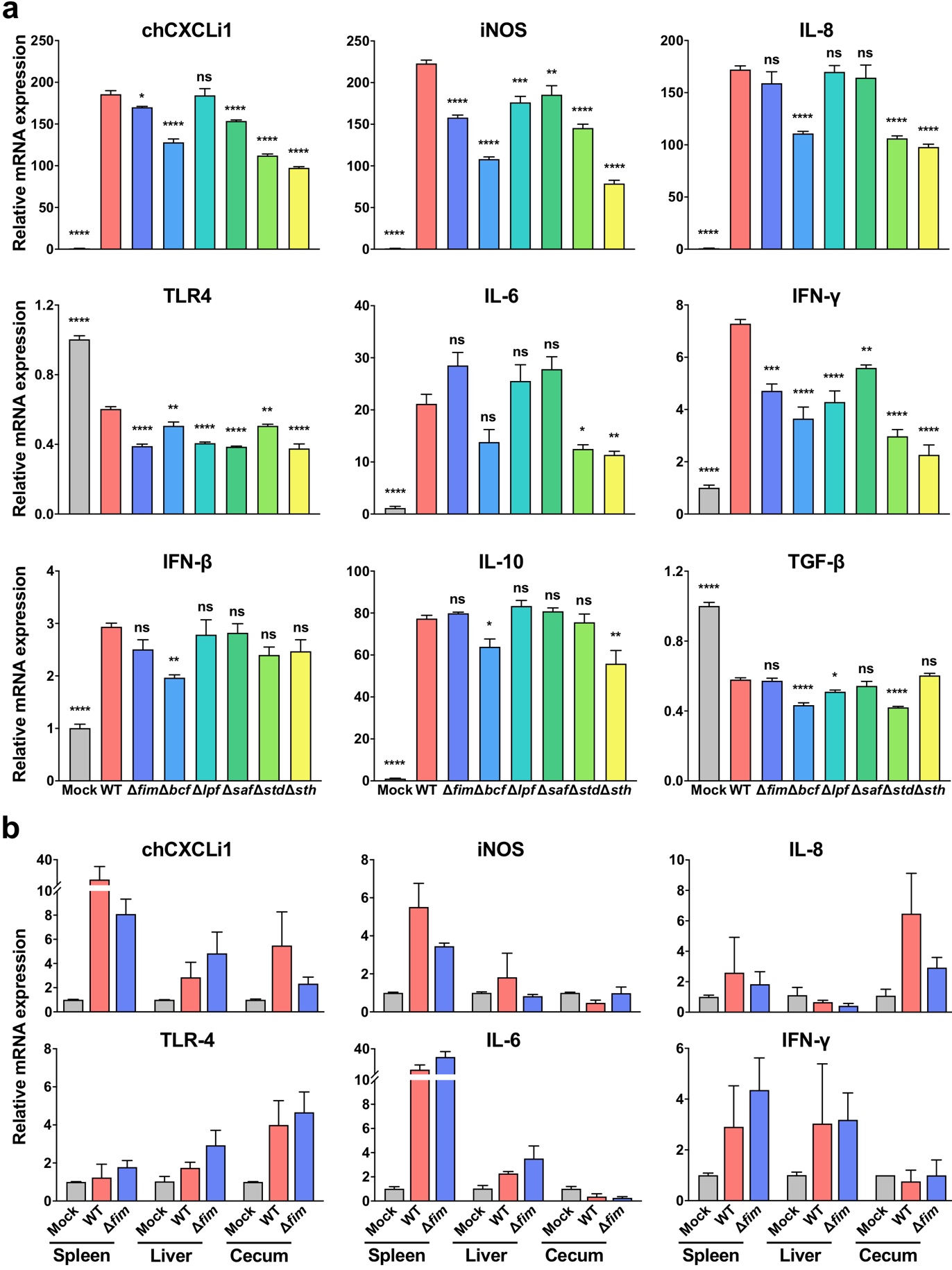


**Supplementary Figure 6. Deletion of fimbriae modulates the mRNA expression of immune-related genes in HD11 (a) and tissues of the 3-day-old chick during the infections (b).** The significant differences between wild-type and other groups are shown above the columns. The data represent the mean (SEM) of three independent experiments. ns, not significant; *, *P* < 0.05; **, *P* < 0.01; ***, *P* < 0.001; ****, *P* < 0.0001.


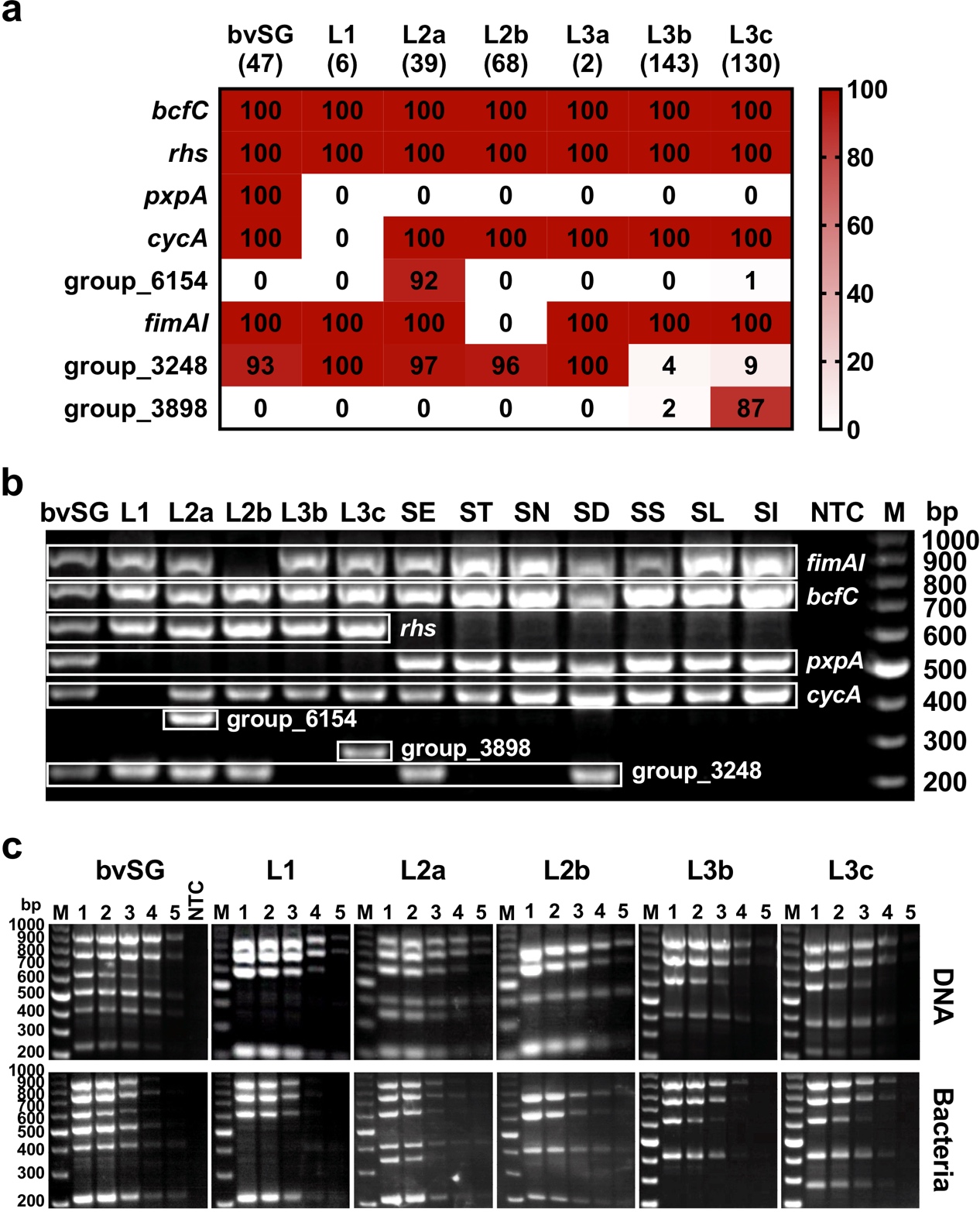


**Supplementary Figure 7. Identification of different sublineages by one-step multiplex PCR. (a)** Specific genes for different sublineages were screened based on pan-genome analysis. The number in each pair of parentheses represents the number of strains tested. The color of individual cells varies with the percentage of gene carriage shown in the cells. **(b)** Multiplex PCR panel of different sublineages. bvSG and other seven serovars are included as controls, including *S*. Enteritidis (SE), *S*. Typhimurium (ST), *S*. Newport (SN), *S*. Dublin (SD), *S*. Senftenberg (SS), *S*. London (SL), and *S*. Indiana (SI). Identical target bands are framed together, with the name of the target gene labeled on the right side. **(c-d)** Sensitivity of the multiplex PCR method for each sublineage, with a template of DNA sample (10-fold serial dilutions of 30.9 ng DNA from lane 1 to lane 5) **(c)** and bacteria (10-fold serial dilutions of 10^6^ CFU bacteria from lane 1 to lane 5) **(d)**. NTC: negative control, M: DNA marker.

**SUPPLEMENTARY TABLES**

Data are presented as an excel file.

**Supplementary Table 1. Information on 320 bvSP isolates originated from China.**

**Supplementary Table 2. Information on isolates for phylogenetic tree construction.**

**Supplementary Table 3. Information on chicken embryo infection assay for different lineages of bvSP.**

**Supplementary Table 4. Information on calculation of invasiveness index for different populations.**

**Supplementary Table 5. Information on desiccation, acid and alkali stress assays.**

**Supplementary Table 6. Information on biofilm formation assay.**

**Supplementary Table 7. Information on biochemical assays.**

**Supplementary Table 8. Data on poultry farming in China.**

**Supplementary Table 9. The carriage of specific genes in different sublineages.**

**Supplementary Table 10. Primers used for construction of fimbrial gene deletion strains.**

**Supplementary Table 11. Primers used for mutiplex PCR, with targeted DNA and amplicon sizes.**

**Supplementary Table 12. Bacterial strains used to confirm the specificity of the multiplex PCR assay.**

**Supplementary Table 13.** **List of tested strains in PCR assay.**

**Supplementary Table 14. Primers used for qPCR.**

**REFERENCES**

1. Bolger AM, Lohse M, Usadel B. Trimmomatic: a flexible trimmer for Illumina sequence data. *Bioinformatics* 2014;**30**:2114–20.

2. Bankevich A, Nurk S, Antipov D *et al.* SPAdes: A New Genome Assembly Algorithm and Its Applications to Single-Cell Sequencing. *Journal of Computational Biology* 2012;**19**:455–77.

3. Yoshida CE, Kruczkiewicz P, Laing CR *et al.* The Salmonella In Silico Typing Resource (SISTR): An Open Web-Accessible Tool for Rapidly Typing and Subtyping Draft Salmonella Genome Assemblies. Hensel M (ed.). *PLoS ONE* 2016;**11**:e0147101.

4. Antipov D, Hartwick N, Shen M *et al.* plasmidSPAdes: assembling plasmids from whole genome sequencing data. *Bioinformatics* 2016:btw493.

5. Alcock BP, Raphenya AR, Lau TTY *et al.* CARD 2020: antibiotic resistome surveillance with the comprehensive antibiotic resistance database. *Nucleic Acids Research* 2019:gkz935.

6. Lim HJ, Lee E-H, Yoon Y *et al.* Portable lysis apparatus for rapid single-step DNA extraction of *Bacillus subtilis*. *J Appl Microbiol* 2016;**120**:379–87.

7. Croucher NJ, Page AJ, Connor TR *et al.* Rapid phylogenetic analysis of large samples of recombinant bacterial whole genome sequences using Gubbins. *Nucleic Acids Research* 2015;**43**:e15–e15.

8. Nguyen L-T, Schmidt HA, von Haeseler A *et al.* IQ-TREE: A Fast and Effective Stochastic Algorithm for Estimating Maximum-Likelihood Phylogenies. *Molecular Biology and Evolution* 2015;**32**:268–74.

9. Letunic I, Bork P. Interactive Tree Of Life (iTOL) v5: an online tool for phylogenetic tree display and annotation. *Nucleic Acids Research* 2021;**49**:W293–6.

10. Wheeler NE, Gardner PP, Barquist L. Machine learning identifies signatures of host adaptation in the bacterial pathogen Salmonella enterica. Didelot X (ed.). *PLoS Genet* 2018;**14**:e1007333.

11. Van Puyvelde S, Pickard D, Vandelannoote K *et al.* An African Salmonella Typhimurium ST313 sublineage with extensive drug-resistance and signatures of host adaptation. *Nat Commun* 2019;**10**:4280.

12. Crawford RW, Gibson DL, Kay WW *et al.* Identification of a Bile-Induced Exopolysaccharide Required for *Salmonella* Biofilm Formation on Gallstone Surfaces. *Infect Immun* 2008;**76**:5341–9.

13. Feng Z, El Hag M, Qin T *et al.* Residue L193P Mutant of RpoS Affects Its Activity During Biofilm Formation in Salmonella Pullorum. *Front Vet Sci* 2020;**7**:571361.

14. Seemann T. Prokka: rapid prokaryotic genome annotation. *Bioinformatics* 2014;**30**:2068–9.

15. Page AJ, Cummins CA, Hunt M *et al.* Roary: rapid large-scale prokaryote pan genome analysis. *Bioinformatics* 2015;**31**:3691–3.

16. Yue M, Rankin SC, Blanchet RT *et al.* Diversification of the Salmonella Fimbriae: A Model of Macro- and Microevolution. Hensel M (ed.). *PLoS ONE* 2012;**7**:e38596.

17. Jiang Y, Chen B, Duan C *et al.* Multigene Editing in the Escherichia coli Genome via the CRISPR-Cas9 System. Kelly RM (ed.). *Appl Environ Microbiol* 2015;**81**:2506–14.

18. Kang X, Zhou X, Tang Y *et al.* Characterization of Two-Component System CitB Family in Salmonella Pullorum. *IJMS* 2022;**23**:10201.

19. Chen J, Zhou X, Tang Y *et al.* Characterization of Two-component System CitB family in Salmonella enterica serovar Gallinarum biovar Gallinarum. *Veterinary Microbiology* 2023:109659.

20. Shah DH, Casavant C, Hawley Q *et al.* *Salmonella* Enteritidis Strains from Poultry Exhibit Differential Responses to Acid Stress, Oxidative Stress, and Survival in the Egg Albumen. *Foodborne Pathogens and Disease* 2012;**9**:258–64.

21. Zhu C, Yue M, Rankin S *et al.* One-Step Identification of Five Prominent Chicken Salmonella Serovars and Biotypes. Fenwick BW (ed.). *J Clin Microbiol* 2015;**53**:3881–3.
